# Not all Online Sexual Activities Are the Same

**DOI:** 10.3389/fpsyg.2019.00339

**Published:** 2019-02-26

**Authors:** Juan Ramón Barrada, Paula Ruiz-Gómez, Ana Belén Correa, Ángel Castro

**Affiliations:** Department of Psychology and Sociology, Universidad de Zaragoza, Teruel, Spain

**Keywords:** online sexual activities, cybersex, pornograhpy, offline sexual behavior, psychosexual well-being, university students

## Abstract

Young people's use and participation in online sexual activities (OSA) has increased in the past two decades and has changed their behavior in the area of sexuality. The existing literature has some important limitations, concerning the assessment of the construct and its orientation toward problematic use, while ignoring its healthy use or social participation and its relationship with well-being. The main objective of this study was to analyze the relationships between the three types of OSA (compulsive, isolated, and social) proposed by Delmonico and Miller, as well as offline sexual behavior, and psychosexual well-being. It was also necessary to evaluate the factor structure of the Internet Sexual Screening Test (ISST). Participants were 1,147 university students of both sexes, aged between 18 and 26 years, who completed a battery of online questionnaires. The main finding of the study is that, when controlling for other online sexual behavior, different types of OSA evaluated relate differently to offline sexual behavior and to psychosexual well-being, and that most young people made healthy use and participation of OSA. It also presents a new structure of the ISST. The discussion emphasizes the need to recognize the positive consequences of OSA to implement programs for the promotion of sexual health.

## Introduction

Internet allows a wide range of behaviors that involve sexual content, topics, and stimuli. These behaviors have been labeled as online sexual activities (OSA; i.e., Shaughnessy et al., 2017). OSA include a large variety of behaviors, both solitary (i.e., viewing pornography) and shared (i.e., sexting).

A construct related to OSA is that of cybersex. Cybersex was defined by Cooper et al. (2004) as the use of the Internet for sexual gratification, so cybersex and OSA could be considered as interchangeable to a large degree. The same idea is expressed by Wéry and Billieux (2017). Nevertheless, Daneback et al. (2005) stated that cybersex was a subcategory of OSA and defined it as the engagement of two or more people in sexual talk while online for the purposes of sexual pleasure. For conceptual clarity and given the polysemy of cybersex, in the rest of the manuscript, we will refer to OSA.

Nowadays, OSA have become a common practice among college students, due to its low cost and easy access, as well as the variety of activities and the anonymity it offers (Ballester-Arnal et al., 2017; Giordano and Cashwell, 2017). In fact, various studies performed in university population have found high levels of OSA use, near 75% (Shaughnessy et al., 2011b, 2017; Döring et al., 2015).

OSA research presents some limitations, possibly due to its being a recent phenomenon and to the variety of activities that it may include (Döring, 2009; Shaughnessy et al., 2011a). This difficulty implies problems and disagreements about how to evaluate it (Eleuteri et al., 2014). Although part of the research points out that the use of the Internet for sexual purposes is a multidimensional phenomenon (Delmonico and Miller, 2003; Pawlikowski et al., 2014), its diverse forms have not usually been evaluated nor have the types of OSA been differentiated when appraising its relationship with other variables.

## Types of OSA Use and Participation

There are some classifications of modalities and types of OSA use. The most widely used classification, especially in clinical terms, is the one that distinguishes between compulsive use (characterized by the lack of control and the risk of leading to addictive behaviors that can affect the daily life of the individual) and non-compulsive use (Cooper et al., 2000). Reviews calculate the prevalence of compulsive use in percentages ranging from 5.6 to 12.9% of the participants (Wéry and Billieux, 2017). A problem with this distinction is that it can confuse non-cumpulsive use with controlled use of OSA and with no use at all of OSA.

Other authors propose different classifications. Shaughnessy et al. (2017) distinguished non-arousal (accessing sexual health information), solitary-arousal (viewing pornography), and partnered-arousal (sending sexually explicit messages). Although we acknowledge that this area of research may be facing a “conceptual chaos” (Wéry and Billieux, 2017, p. 239), we will follow the model of Delmonico and Miller (2003), who mentioned three main types. The first one, and the most studied, is compulsive use, with the already mentioned features. Despite being the least prevalent of all three, it is the one with the most negative consequences for individuals (Wéry and Billieux, 2017). Most users do not present problematic use, which raises the need to investigate other uses of OSA (Döring et al., 2015).

The second element referred to by Delmonico and Miller (2003) is isolated use, performed by individuals who do not wish to maintain interaction with other people. The most studied isolated activity is the viewing of pornography, which is common in young people worldwide (Séguin et al., 2018; Willoughby et al., 2018).

The third category is social participation, carried out by people who want an online interaction with other individuals, for example, through sexual chats. This section would include sexting, a common practice at present among adolescents and young people (Ybarra and Mitchell, 2014; Doornwaard et al., 2017). Social OSA use can have positive consequences for the individual, because it offers opportunities to gain sexual experience (Döring, 2009), meet potential new partners, and increase social networks (Whitty, 2008), or for the personal development of members of sexual minorities, who find a way to express themselves on the Internet (Poon et al., 2005).

## Correlates of OSA

Sexuality on the Internet differs as a function of individual's gender, sexual orientation, and relationship status (Döring, 2009). Most studies conclude that men participate in more OSA than women, regardless of the type of activity (Ballester-Arnal et al., 2017), although it seems that women are more interested in social activities like sexting, and men in isolated activities, such as viewing pornography (Delmonico and Miller, 2003; Ballester-Arnal et al., 2010; Shaughnessy et al., 2011b; Wéry and Billieux, 2017). In terms of sexual orientation, the literature has usually focused on heterosexual individuals and couples, and has not evaluated the sexual scripts of sexual minorities (Courtice and Shaughnessy, 2017). There is a consensus that sexual minority people use more OSA; especially, they participate in more social activities (e.g., sexual chats, dating apps) than heterosexual individuals because Internet is one of their main resources for finding a partner (Ross and Kauth, 2002; Chaney and Blalock, 2006).

Some studies have also concluded that online sex is used not only by people without a romantic relationship, but also, many people use it as a complement to their offline relationship (Griffiths, 2012). In fact, currently, many young people's romantic relationships originated in online contact (Daneback et al., 2005), which shows the relationship between online and offline sexual behavior. Some authors have concluded that some individuals are more sexually active regardless of the kind of sex they practice (Daneback et al., 2005; Griffiths, 2012). Online sex has habitually been related to offline sex, especially from a negative viewpoint, concluding that the individuals who practice more OSA perform more risky sexual behaviors, such as having sex with a large number of partners or using condoms inconsistently (Liau et al., 2006).

There is some literature on the relationship between online sex and psychological well-being. Many studies have offered a negative view of OSA, focused on compulsive use and finding direct relationships with anxiety, depression, low self-esteem, loneliness, or relational difficulties (Kor et al., 2014; Harper and Hodgins, 2016). However, associations have also been found between isolated activities (viewing pornography) and poorer psychological functioning (Harper and Hodgins, 2016), as well as between social OSA (sexting), relational anxiety, and lower self-esteem (Ybarra and Mitchell, 2014; Weisskirch et al., 2017).

Despite this negative view of the relationship between OSA and psychological functioning, many works have focused on studying specific well-being in the area of sexuality. The existing works have studied, on the one hand, the sexual functioning of online sex users, and on the other, its relationship with variables such self-esteem as a sexual partner or sociosexuality. In terms of sexual performance, it was found that compulsive sex online users had poorer sexual functioning, in particular, a decrease in satisfaction and interest in sex, as well as erectile dysfunction (Voon et al., 2014).

Regarding the other mentioned variables, Kvalem et al. (2014) found a direct relation between viewing pornography and sexual self-esteem in males. Other authors, like Zheng and Zheng (2014) and Shaughnessy and Byers (2013), evaluated the relation between use of online sex and sociosexuality, understood as the willingness to engage in sexual activity outside a committed relationship (Penke and Asendorpf, 2008). It would be interesting to continue examining these relations and to complete the results of these studies that indicate that people who use more OSA, especially social OSA, tend to have more unrestricted sociosexuality, that is, they will have more casual sexual partners (Daneback et al., 2005). Due to the current relevance of OSA and its possible relation with psychosexual well-being, it was considered relevant to investigate in this direction.

## Measurement of OSA

One of the main limitations of the existing literature on OSA is its evaluation. Evaluation tools have sprung up in the last two decades, but many of them are not validated, so their psychometric properties and usefulness are unknown. Those that are validated present some problems (Eleuteri et al., 2014; Wéry and Billieux, 2017). A large part of the proposed instruments is focused on problematic use (Hook et al., 2010; Eleuteri et al., 2014).

As Wéry and Billieux (2017) noted, one of the most used questionnaires is the Internet Sex Screening Test (ISST; Delmonico and Miller, 2003). The ISST is a 25-item questionnaire, with a yes/no response format. Item wording can be seen in Table 1. The main advantage of the ISST is that it measures several dimensions of OSA. Delmonico and Miller (2003) described five different factors and two single-item scales. The three main factors (by number of items and by presenting Cronbach's alphas over 0.70) were Compulsive use (six items), Social participation (five items), and Isolated use (three items).

**Table 1:** Item loadings of the original and extended version of the Internet Sex Screening Test and item descriptives.

**Item loadings**	**COM**	**ISO**	**SOC**	**Prop**
1. I have some sexual sites bookmarked.	0.04/0.30	**0.72/0.53**	−0.01/0.07	0.28
×2. I spend more than 5 h per week using my computer for sexual pursuits.	**0.39/0.60**	**0.44**/0.27	0.01/−0.01	0.05
3. I have joined sexual sites to gain access to online sexual material.	**0.55/0.73**	0.29/0.03	0.20/0.14	0.03
×4. I have purchased sexual products online.	**0.35/0.43**	0.00/−0.22	**0.33**/0.24	0.09
5. I have searched for sexual material through an Internet search tool.	−0.11/0.10	**0.71/0.52**	0.03/0.15	0.68
*6. I have spent more money for online sexual material than I planned.	**1.00**/ **1.06**	−0.18/–**0.38**	0.18/0.01	0.02
×7. Internet sex has sometimes interfered with certain aspects of my life.	**0.38/0.50**	**0.31**/0.20	−0.07/−0.02	0.11
×8. I have participated in sexually related chats.	0.05/0.19	**0.42**/0.06	**0.43/0.54**	0.18
9. I have a sexualized username or nickname that I use on the Internet.	0.27/**0.45**	**0.35**/0.11	0.21/0.19	0.12
×10. I have masturbated while on the Internet.	**−0.31**/−0.02	**1.12/0.87**	−0.00/0.28	0.63
11. I have accessed sexual sites from other computers besides my home.	0.03/0.26	**0.59/0.35**	0.14/0.20	0.20
12. No one knows I use my computer for sexual purposes.	−0.08/0.02	**0.49/0.50**	−0.25/−0.09	0.28
13. I have tried to hide what is on my computer or monitor so others cannot see it.	0.03/0.28	**0.74/0.62**	−0.13/−0.05	0.47
14. I have stayed up after midnight to access sexual material online.	−0.09/0.18	**0.80/0.59**	0.02/0.12	0.27
*15. I use the Internet to experiment with different aspects of sexuality (e.g., bondage, homosexuality, anal sex, etc.)	0.07/**0.31**	**0.68/0.48**	0.02/0.10	0.25
16. I have my own website which contains some sexual material.	**0.71/0.81**	0.10/−0.02	−0.04/−0.07	0.03
×^*^17. I have made promises to myself to stop using the Internet for sexual purposes.	**0.85/0.84**	0.01/0.14	**−0.56/–0.48**	0.08
*18. I sometimes use cybersex as a reward for accomplishing something (e.g., finish a project, stressful day, etc.)	0.25/**0.45**	**0.54/0.37**	−0.04/0.05	0.09
×^*^19. When I am unable to access sexual information online, I feel anxious, angry, or disappointed.	**0.40/0.58**	**0.45/0.34**	−0.14/−0.12	0.10
×^*^20. I have increased the risks I take online (give out name and phone number, meet people offline, etc.)	**0.37/0.52**	0.25/−0.11	**0.45/0.44**	0.08
*21. I have punished myself when I use the Internet for sexual purposes (e.g., time-out from computer, cancel Internet subscription, etc.)	**0.98**/ **1.08**	0.00/−0.02	−0.27/**–0.36**	0.03
×22. I have met face to face with someone I met online for romantic purposes.	−0.01/0.14	**0.34**/−0.08	**0.62/0.66**	0.18
×23. I use sexual humor and innuendo with others while online.	−0.00/0.18	**0.47**/0.03	**0.57/0.66**	0.21
×^*^24. I have run across illegal sexual material while on the Internet.	0.17/0.24	−0.09/−0.18	0.18/0.05	0.21
25. I believe I am an Internet sex addict.	**0.74**/**0.88**	0.25/0.05	0.01/0.03	0.02
*26. I like to use Skype or other similar applications for sexual purposes.	**–/0.32**	–/0.01	**–/0.69**	0.06
27. I like to use WhatsApp or other similar applications for sexual purposes (chats, sending photos, videos, etc.).	–/0.01	–/0.05	**–/0.80**	0.14
Interfactor correlations	COM	ISO	SOC	
COM				
ISO	0.60/0.43			
SOC	0.33/0.52	0.10/0.29		

COM, Compulsive use; ISO, Isolated use; SOC, Social use; Prop, proportion of participants answering ‘yes’ to the item. Cells with two values present loadings or interfactor correlations for the original and extended version, in that order. Bold values correspond to loadings greater than |0.30|. Items with “ × ” and “*” indicate problematic items due to two loadings over |0.30| or no loading over that value in the original and extended version, in that order. Underlined proportions indicate extreme values, in the range [0, 0.20] or [0.80, 1].

A single validation study has been conducted with the ISST (Ballester-Arnal et al., 2010). Delmonico and Miller (2003) included a reference to a “manuscript submitted for publication” of a validation study, but we have not been able to locate it. Ballester-Arnal et al. (2010) validated the Spanish version with a sample of university students. There were several important limitations in the assessment of the internal structure of the ISST responses in that study. First, the authors used principal component analysis, which is not a factor analysis technique. Second, they used the screen test and the Kaiser–Cattell rule to determine the number of items to retain, options that, arguably, are not the best available (Izquierdo et al., 2014). Third, and most important, they analyzed the product-moment correlations matrix, an incorrect approach, considering that the items are dichotomous (Bandalos and Finney, 2010). Ballester-Arnal et al. (2010) identified five factors. The three most important (again, by number of items and by presenting Cronbach's alpha over 0.70) were the same as those reported by Delmonico and Miller (2003): Compulsive use (now with eight items), Social participation (six items), and Isolated use (six items). The overlap in the distribution of items by factor is far from perfect in the two versions.

An additional problem of the ISST is that it was developed more than 15 years ago. In this time, the way Internet is used has changed. Things that were very difficult at the beginning of the century due to bandwidth limitations, like video-chatting, can now be easily performed with a mobile-phone. This implies that some current practices of online sex behavior are not covered by the ISST items.

Thus, the present situation is not satisfactory. Researchers must choose between using questionnaires mainly focused on problematic or compulsive use of OSA (thus, missing relevant aspects of online sex behavior), assessing key variables such as pornography use with a single item (with unknown reliability, but expectedly lower than with multiple items; i.e., Grubbs et al., 2017, 2018), or using a questionnaire with unknown psychometric properties (such as the ISST).

## The Present Study

Therefore, it seems that the existing literature on OSA has some important limitations. First, it has focused on compulsive or problematic behavior, paying less attention to other non-problematic uses and to the multidimensional nature of these activities. Second, the relationships between different types of OSA, offline sexual behavior, and especially, psychosexual well-being have not received much attention. And third, the assessment of the construct has not been clear and has failed to differentiate between the different dimensions of uses. For this reason, this study, while offering the first rigorous validation of the ISST and improving this instrument, has the goal of analyzing the relationships between the three contemplated modalities of OSA use (compulsive, isolated, and social), offline sexual behavior (vaginal, anal, and masturbatory), and the evaluated psychosexual variables.

## Materials and Methods

### Participants

The initial sample was made up of 2,097 participants aged between 18 and 63 years (*M* = 21.93, *SD* = 4.01). The survey platform used (based on LimeSurvey) created a new row in the database with every new access to the survey, independently of whether or not any question was answered. In this initial recount, we are considering as participants those who, at least, provided responses about their sex and age. Three inclusion criteria were employed: (1) to be currently studying at the university (180 participants excluded); (2) to be between 18 and 26 years old, according to the most common age range in Spanish university students (115 participants excluded), and (3) to present two or fewer missing items on the extended version of the ISST (Delmonico and Miller, 2003; 655 participants excluded, with 597 not responding to any ISST item). With the first and second inclusion criteria, we expected to create a more homogenous sample, with a clearer definition of the population of study. The second criterion allowed us to remove outliers in terms of age and potential problems of a mixture of different populations, as it can be expected that students in the age range of [18, 26] and older students probably differ in many critical variables. By selecting this age range, we continued the criterion of previous studies (Correa et al., 2017). We decided to remain consistent across studies in order to reduce researchers' degrees of freedom and, thus, avoid potential *p*-hacking (Wicherts et al., 2016). The large drop of participants due to the third inclusion criterion can be explained by the fact that ISST was the last instrument of a large battery of instruments, so we do not expect participants' fatigue to be correlated to any relevant variable of the study.

After applying these criteria, the final sample comprised 1,147 university students (70% female, 30% male), aged between 18 and 26 (*M* = 21.08, *SD* = 2.00). Of these participants, 87.7% described themselves as heterosexual, 6.2% as bisexual, 4.7% as homosexual, and 1.4% as other orientations. Due to the small sample sizes of non-heterosexual participants, the participants were grouped into heterosexual individuals (87.7%), and sexual minority people (12.3%). Of the participants, 59.5% had a partner at the time of the study. Part of the present sample overlaps the sample used in Correa et al. (2017), but, in that study, sexual minority participants were excluded, and other variables and research questions were considered (sociodemographic and psychosexual characteristics of students who engage in casual sex).

### Instruments

#### Sociodemographic and Sexual Behavior Questionnaire

We used a questionnaire employed in previous studies (Castro and Santos-Iglesias, 2016; Correa et al., 2017). We asked about sex/gender (men, women), age, sexual orientation (heterosexual, homosexual, bisexual, other), and relational status. We also asked about lifetime sexual behavior (age at the first sexual intercourse, number of partners), sexual behavior in the previous 3 months (number of partners, number of relations, number of relations with condom, and number of relations under the influence of alcohol and drugs), both for vaginal and anal behavior. We also asked about frequency of masturbation.

#### Internet Sex Screening Test (ISST; Delmonico and Miller, 2003)

As previously described, the ISST is composed of 25 items with dichotomous response. Whereas in the original version, the response options were 0 = *No* and 1 = *Yes*, in the Spanish version (Ballester-Arnal et al., 2010), they are 0 = *False* and 1 = *True*. As previously discussed, the internal structure of the ISST is not clear. We used the Spanish adaptation of Ballester-Arnal et al. (2010). In order to update the ISST content to include newer practices in OSA, two additional items were included: (a) “I like to use Skype or other similar applications for sexual purposes” and (b) “I like to use WhatsApp or other similar applications for sexual purposes (chats, sending photos, videos, etc.).”

#### Short Version (Wiederman and Allgeier, 1993) of the Sexuality Scale (SSS; Snell and Papini, 1989)

This instrument has 15 items that assess perceptions of one's own sexuality through three components: Self-esteem as a Sexual Partner (e.g., “I am a good sexual partner”), Dissatisfaction with Sexual Life (e.g., “I'm depressed about the sexual aspects of my life”), and Sexual Preoccupation (e.g., “I'm constantly thinking about having sex”). It is rated on a five-point Likert-type scale ranging from 1 = *strongly disagree* to 5 = *strongly agree*. In this study, we obtained Cronbach's alphas of 0.89 for Self-Esteem as a Sexual Partner (95% CI [0.88, 0.90]); 0.88 for Dissatisfaction with Sexual Life (95% CI [0.86, 0.89]); and 0.86 for Sexual Preoccupation (95% CI [0.84, 0.88]). We used the Spanish adaptation of Soler et al. (2016).

#### Sociosexual Orientation Inventory-Revised (SOI-R; Penke and Asendorpf, 2008)

This instrument has nine items that assess sociosexual orientation on the basis of three dimensions: Behavioral (e.g., “With how many different partners have you had sexual intercourse without having an interest in a long-term committed relationship with this person?”), Attitudinal (e.g., “Sex without love is OK.”), and Desire (e.g., “How often do you have fantasies about having sex with someone with whom you do not have a committed romantic relationship?”). These items are rated on a nine-point scale, ranging from 1 = *0* to 9 = *20 or more* in the Behavioral factor; from 1 = *strongly disagree* to 9 = *strongly agree* in the Attitudinal factor; and from 1 = *never* to 9 = *at least once a day* in the Desire factor. Due to an error in the transcription of the questionnaire, the items corresponding to the Attitudinal factor were rated on a seven-point scale, with the same anchors as the original scale. Cronbach's alphas obtained in this study were 0.84 for the Behavioral factor (95% CI [0.82, 0.87]); 0.79 for the Attitudinal factor (95% CI [0.76, 0.81]); and 0.84 for the Desire factor (95% CI [0.82, 0.86]). We used the Spanish validation of Barrada et al. (2018).

#### Arizona Sexual Experience Scale (ASEX; McGahuey et al., 2000)

The ASEX is composed of five items that assess five basic areas of sexual functioning in men and women regardless of their sexual orientation: desire, arousal, erection/vaginal lubrication, ability to reach orgasm, and satisfaction with orgasm. The male and female versions differ on the third question. Items are rated on a scale with six response options, ranging from 1 = *hyperfunction* to 6 = *hypofunction*. Higher scores indicate greater sexual dysfunction. Cronbach's alpha obtained in this study was 0.73 (95% CI [0.70, 0.76]). We used the Spanish validation of Santos-Iglesias et al. (2017).

### Procedure

The data were collected on-line. We approached the participants through the e-mail distribution lists of the university. Each student registered on the lists whose administrators gave access to the corresponding information received an e-mail explaining the goal of the study, contact information of the principal investigator, participation conditions, and a link to access the survey. Only those who gave their online informed consent, included at the beginning of the survey, could gain access. After completing the survey, the participants stated whether they wanted to participate in the draw of an iPad Mini™. Those who responded affirmatively provided their name and e-mail. This study and its protocol were carried out in accordance with the recommendations of the ethics research committee of the region. All subjects gave written informed consent in accordance with the Declaration of Helsinki.

### Data Analysis

Firstly, six indexes were generated to assess risky sexual behavior, three related to vaginal sex and three to anal sex. To calculate them, we divided the number of relations with condom/under the influence of alcohol/under the influence of drugs in the previous 3 months by the total number of relations in the previous 3 months. Therefore, the range of possible index values was between 0 and 1. While higher scores in the condom use index indicated lower risk, higher scores in the alcohol or drug index indicated higher risk.

Secondly, given the previously noted problems with the internal structure of the ISST, we performed an exploratory factorial analysis (EFA). In order to determine the numbers of dimensions to be retained—which could not be anticipated before data analysis—, we used parallel analysis (Garrido et al., 2013). As we employed dichotomous items, models were analyzed using robust weighted least squares (WLSMV estimator in M*Plus*). Goodness of fit of all the derived models was assessed with the common cut-off values for the fit indices (Hu and Bentler, 1999). A comparative fit index (CFI) and Tucker-Lewis index (TLI) with values >0.95 and a root mean square error of approximation (RMSEA) of <0.06 were indicative of a satisfactory fit. It should be noted that those cut-offs were developed for confirmatory factor analysis with continuous responses, so the values should be considered with caution. The authors are not aware whether specific cut-offs have been proposed for EFAs with categorical variables. Items were marked as problematic if they presented more than one unique loading or no loading over |0.30|. We used ‘problematic’ to indicate items that would lead to doubts about how to score them if dimension scores are based on the sum of observed scores.

We tested models with the original 25-item version and the extended 27-item version. The solutions for the two models were compared with the congruence coefficient. The congruence coefficient is an index of similarity between factors that has boundaries of −1 and +1. A congruence coefficient in the range of 0.85–0.94 corresponds to a fair similarity between factors, whereas a coefficient of 0.95 or higher indicates a good level of similarity, such that the factors can be considered equal (Lorenzo-Seva and Ten Berge, 2006).

Thirdly, we computed the proportion of true-responses in the ISST items. As the responses can only be 0 or 1, extreme means—in the range [0, 0.20] or [0.80, 1]—will be considered as problematic. We used “problematic” for those items, as extreme means imply low variances, which reduces their value to assess individual differences.

Fourthly, we computed scores for the different OSA dimensions. Given the small number of items per dimension, the low average score in each item, the dichotomous nature of the response scale, and the presence of relevant cross-loadings (see results below), we considered it more appropriate to use standardized factor scores instead of observed scores. Factor scores are the weighted sums of observed variables, with the weights based on item loadings (Grice, 2001). With factor scores in an EFA context, all items provide information for the computation of scores of all the factors, although their contribution varies by item and factor. While a complex factorial structure implies problems to compute total scores based on the sum of observed responses, this is not the case with factor scores. We computed conditional reliabilities for these scores with the equations provided by Nicewander (2018) based in test information functions.

Fifthly, we computed the associations of the different dimensions of OSA with the rest of the variables. The effect sizes for the sociodemographic variables (sex, sexual orientation, being involved in a relationship) were Cohen's *d*; for vaginal sexual behavior, anal sexual behavior, masturbation, and psychosexual variables, we calculated product-moment correlations. We computed 95% confidence intervals for these associations.

Finally, we performed hierarchical regressions to evaluate the association of the different dimensions of OSA while controlling for the other dimensions. In the first block, we introduced the sociodemographic variables; in the second block, the OSA scores; and as dependent variables, those related to sexual behavior and psychosexual variables.

Following Wilkinson and The Task Force on Statistical Inference (1999), we chose the metric of variables that could better communicate our results. When the units of measurement were meaningful on a practical level (sexual behavior: age in years of sexual initiation, for instance), we used the original metric of the variables. For scale scores with a metric that is more difficult to interpret, we standardized the dependent variables before the regression.

For the EFA analyses, we used Mplus 7.4 (Muthén and Muthén, 1998-2015) and R 3.5.2 (R Core Team, 2018). Factor scores were computed with the option “SAVE = FSCORES” and test information functions with the option “PLOT: TYPE IS PLOT2” of Mplus. For the rest of the analyses, we used SPSS version 20 and R version 3.5.2 (packages *MBESS* 4.4.3 *psych* 1.8.12). Reported confidence intervals for Cronbach's alphas were computed with bias corrected and accelerated bootstrap (Kelley and Pornprasertmanit, 2016).

## Results

### ISST Internal Structure and Reliabilities

**Figure 1:**
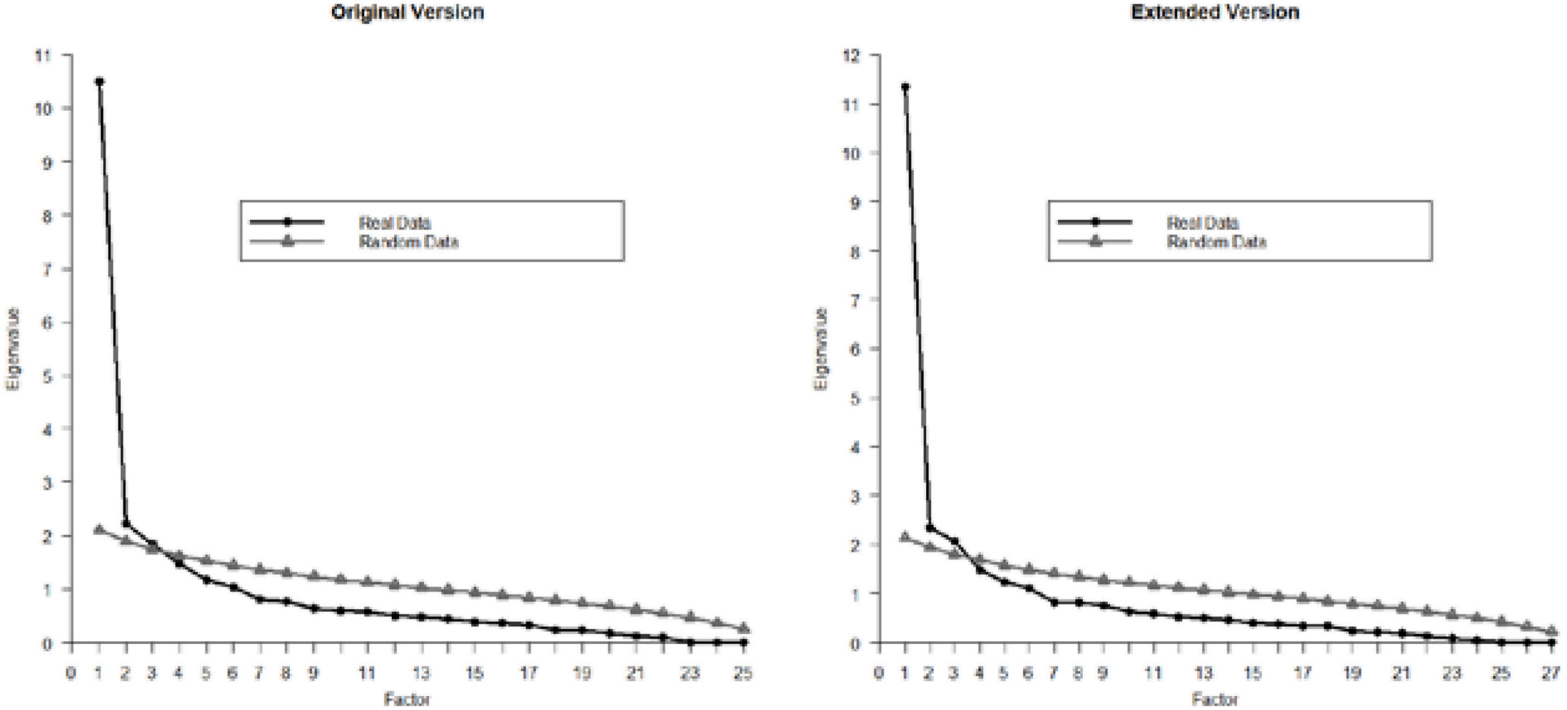
Parallel analysis of the Internet Sex Screening Test responses with the original version (25 items) and extended version (27 items).

The results from the parallel analysis are shown in Figure 1. It can be seen that both in the 25-item and the 27-item versions, three eigenvalues from the sample were greater than the eigenvalues from the randomly generated datasets. The appropriateness of retaining three factors was clearer for the extended version, as the difference between the third sample eigenvalue and the random eigenvalue was larger. For both versions, a three-factor solution offered a satisfactory fit (although the TLI was <0.01 below the cut-off value of 0.95): For the original version, $\chi_{\left(228\right)}^{2}$ = 470.55, CFI = 0.956, TLI = 0.942, RMSEA = 0.030; for the extended version, $\chi_{\left(273\right)}^{2}$ = 541.34, CFI = 0.957, TLI = 0.945, RMSEA = 0.029.

Item loadings and item means are shown in Table 1. In both versions, we interpreted the three recovered dimensions in the same way. The first factor was related to Compulsive use, with items like “I believe I am an Internet sex addict” (loading: λ_extended version_ = 0.78). The second factor could be labeled as Isolated behavior (e.g., “I have masturbated while on the Internet”; λ_extended_ = 0.87). The third factor was related to Social behavior (e.g., “I like to use WhatsApp or other similar applications for sexual purposes [chats, sending photos, videos, etc.]”; λ_extended_ = 0.80). According to the congruence coefficient (cc), the similarity of the loadings in the original and extended versions could be considered as good for the Compulsive factor (cc = 0.95) and fair for the Isolated (cc = 0.91) and Social (cc = 0.93) factors.

Both versions presented an important proportion of items with problematic loadings. In the original versions, 11 items out of 25 (44%) presented no loading or more than a single loading over |0.30|. In the extended version, the same problem was present in 9 out of 27 items (33%). The item “I have run across illegal sexual material while on the Internet” presented no relevant loading in either version.

The mean proportion of True responses in the items was 0.19 and 0.18 in the original and extended versions. Considering the original version, 15 items out of 25 (60%) presented extreme means (in the range of [0, 0.20]); for the extended version, that proportion was 17 out of 27 items (63%). The item with the highest mean was “I have searched for sexual material through an Internet search tool” (*M* = 0.68) and the lowest for the item “I have spent more money for online sexual material than I planned” (*M* = 0.02).

Although the internal structure of both versions was not simple, we consider that the extended version improved the results. First, the proportion of items with relevant cross-loadings was reduced. Second, the Social factor was better defined: In the original version, all the items with relevant loadings on that factor also presented relevant cross-loadings. For instance, the item “I have participated in sexually related chats” (clearly tapping the Social dimension) presented a relevant cross-loading of 0.42 in the Isolated factor in the original version, whereas this loading was reduced to 0.06 in the extended version. For this reason, in the rest of the analyses, we used factor scores derived from the extended version. The product-moment correlation between factor scores of the original and extended versions was 0.99 for the Compulsive factor, 0.95 for the Isolated factor, and 0.77 for the Social factor.

**Figure 2:**
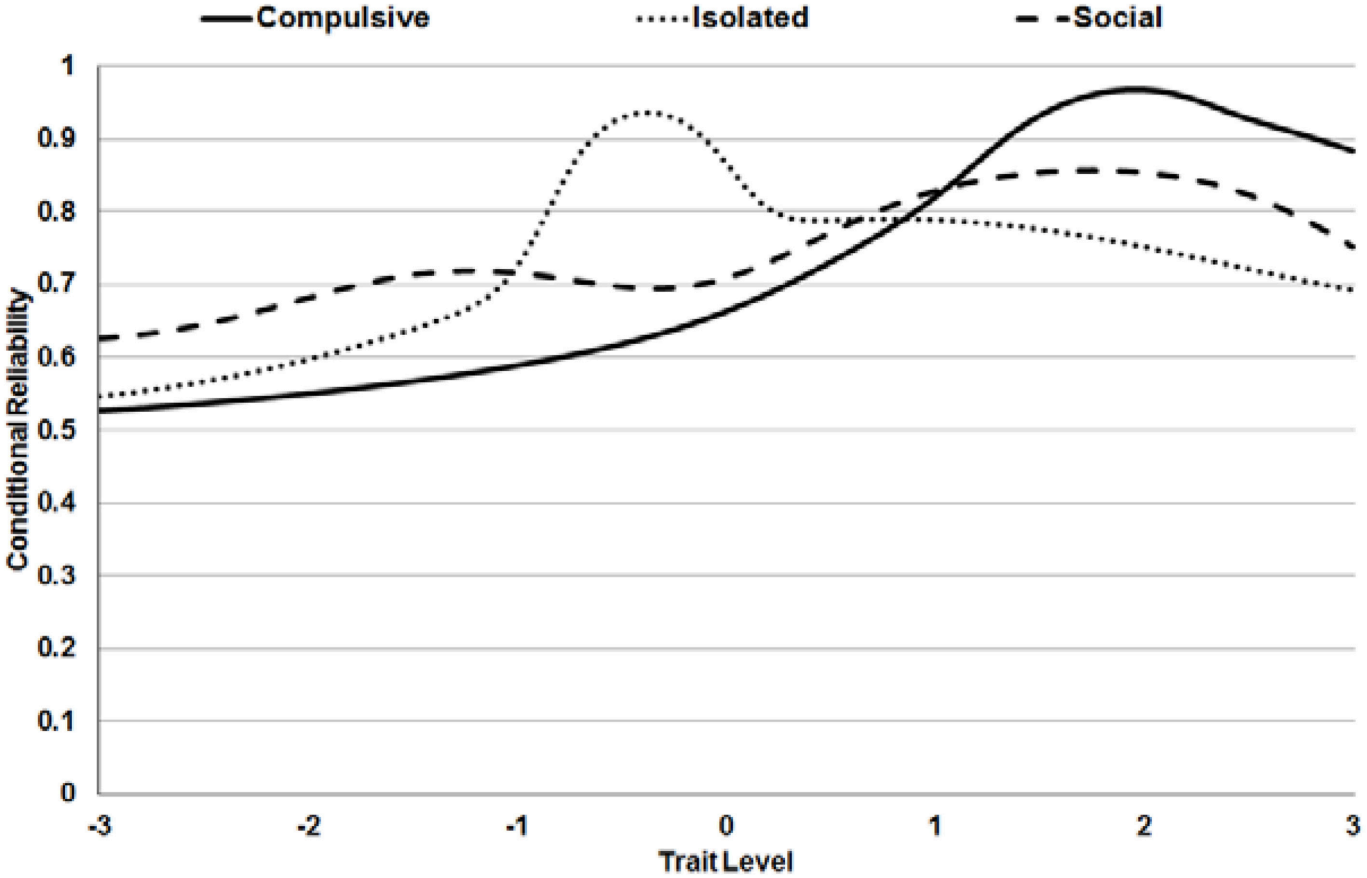
Conditional reliabilities for the scores of the Internet Sex Screening Test (extended version).

Conditional reliabilities are shown in Figure 2. If we consider as appropriate reliability values over 0.70, that cut-off point was surpassed for Compulsive scores over 0.30, Isolated scores in the range [−1.1, 2.9], and Social scores over −0.10. In other words, the measurement was most accurate for medium-high scores.

### Associations of OSA

The associations of OSA with the other measured variables can be seen in Table 2. For simplicity, we will not comment confidence intervals. Those can be seen in the table.

**Table 2:** Associations and their 95% confidence intervals of OSA with sociodemographic variables, vaginal and anal sexual behavior, masturbation, and psychosexual variables.

	**Compulsive**		**Isolated**		**Social**		
	***d* [95% CI]**	***p***	***d* [95% CI]**	***p***	***d* [95% CI]**	***p***	***n***
**SOCIODEMOGRAPHIC**							
Men	**1.17 [1.03, 1.31]**	** <0.001**	**1.21 [1.07, 1.36]**	** <0.001**	**0.68 [0.54, 0.81]**	** <0.001**	1147
Relation	**−0.27 [−0.39**, **−0.14]**	** <0.001**	**−0.22 [−0.35**, **−0.10]**	** <0.001**	**−0.23 [−0.35**, **−0.11]**	** <0.001**	1117
Minority	**0.82 [0.64, 1.01]**	** <0.001**	**0.48 [0.30, 0.66]**	** <0.001**	**0.94 [0.75, 1.12]**	** <0.001**	1134
	***r*** **[95% CI]**	***p***	***r*** **[95% CI]**	***p***	***r*** **[95% CI]**	***p***	***n***
**VAGINAL SEXUAL BEHAVIOR**							
Age of initiation	0.01 [−0.05, 0.07]	0.768	0.06 [−0.01, 0.12]	0.079	**−0.11 [−0.17**, **−0.05]**	** <0.001**	1004
Partners	**0.12 [0.07, 0.18]**	** <0.001**	**0.08 [0.02, 0.13]**	**0.011**	**0.26 [0.21, 0.32]**	** <0.001**	1130
Partners (3 months)	0.05 [−0.00, 0.11]	0.070	0.03 [−0.02, 0.09]	0.248	**0.17 [0.11, 0.22]**	** <0.001**	1125
Relations (3 months)	−0.03 [−0.09, 0.03]	0.337	−0.05 [−0.10, 0.01]	0.119	0.02 [−0.04, 0.08]	0.451	1119
Condom use	−0.04 [−0.11, 0.03]	0.241	0.01 [−0.06, 0.08]	0.747	−0.03 [−0.09, 0.04]	0.474	808
Sex with alcohol	0.05 [−0.02, 0.12]	0.170	0.03 [−0.04, 0.10]	0.351	0.06 [−0.00, 0.13]	0.066	810
Sex with drugs	**0.11 [0.04, 0.17]**	**0.002**	**0.07 [0.01, 0.14]**	**0.033**	**0.14 [0.08, 0.21]**	** <0.001**	819
**ANAL SEXUAL BEHAVIOR**							
Age of initiation	**−0.17 [−0.28**, **−0.05]**	**0.005**	**−0.16 [−0.27**, **−0.04]**	**0.008**	**−0.25 [−0.35**, **−0.13]**	** <0.001**	274
Partners	**0.24 [0.18, 0.29]**	** <0.001**	**0.16 [0.11, 0.22]**	** <0.001**	**0.30 [0.25, 0.35]**	** <0.001**	1123
Partners (3 months)	**0.20 [0.14, 0.25]**	** <0.001**	**0.17 [0.11, 0.22]**	** <0.001**	**0.27 [0.21, 0.32]**	** <0.001**	1121
Relations (3 months)	**0.18 [0.13, 0.24]**	** <0.001**	**0.14 [0.08, 0.2]**	** <0.001**	**0.20 [0.14, 0.25]**	** <0.001**	1121
Condom use	−0.04 [−0.22, 0.14]	0.643	−0.09 [−0.26, 0.10]	0.348	0.01 [−0.17, 0.19]	0.948	118
Sex with alcohol	−0.05 [−0.22, 0.13]	0.616	0.01 [−0.17, 0.19]	0.950	−0.05 [−0.23, 0.13]	0.588	119
Sex with drugs	**0.18 [0.00, 0.35]**	**0.049**	0.17 [−0.01, 0.34]	0.063	0.18 [−0.00, 0.35]	0.052	119
**MASTURBATION**	**0.51 [0.46, 0.55]**	** <0.001**	**0.57 [0.53, 0.61]**	** <0.001**	**0.41 [0.36, 0.46]**	** <0.001**	1105
**PSYCHOSEXUALITY**							
SS self–esteem	−0.02 [−0.08, 0.04]	0.553	0.01 [−0.05, 0.07]	0.630	**0.08 [0.02, 0.14]**	**0.009**	1071
SS dissatisfaction	**0.19 [0.14, 0.25]**	** <0.001**	**0.17 [0.11, 0.22]**	** <0.001**	**0.09 [0.03, 0.15]**	**0.003**	1084
SS preoccupation	**0.35 [0.3, 0.4]**	** <0.001**	**0.29 [0.24, 0.34]**	** <0.001**	**0.3 [0.24, 0.35]**	** <0.001**	1120
SOI–R behavior	**0.22 [0.16, 0.28]**	** <0.001**	**0.14 [0.08, 0.19]**	** <0.001**	**0.39 [0.34, 0.44]**	** <0.001**	1097
SOI–R attitudes	**0.21 [0.15, 0.26]**	** <0.001**	**0.22 [0.16, 0.27]**	** <0.001**	**0.30 [0.25, 0.36]**	** <0.001**	1123
SOI–R desire	**0.43 [0.38, 0.47]**	** <0.001**	**0.38 [0.33, 0.43]**	** <0.001**	**0.41 [0.36, 0.45]**	** <0.001**	1119
ASEX sexual function^a^	**−0.19 [−0.25**, **−0.13]**	** <0.001**	**−0.22 [−0.28**, **−0.17]**	** <0.001**	**−0.18 [−0.23**, **−0.12]**	** <0.001**	1061

*CI = 95% confidence interval. Men is a dummy variable where 0 = woman and 1 = man. Relation is a dummy variable where 0 = not in a romantic relationship and 1 = in a romantic relationship. Minority is a dummy variable where 0 = heterosexual and 1 = sexual minority. COM, Compulsive use; ISO, Isolated use; SOC, Social participation; SS, Sexuality Scale; SOI-R, Sociosexual Orientation Inventory-Revised; ASEX, Arizona Sexual Experience Scale. Bold values correspond to statistically significant results*.

a *In the ASEX, higher scores indicate lower sexual functioning*.

#### Association of OSA and Sociodemographics

Men presented a higher use of online sex than women in all three dimensions, with larger effects for Compulsive (*d* = 1.17) and Isolated (*d* = 1.21) than for the Social (*d* = 0.68) factor. Individuals engaged in a romantic relationship used sex online to a lesser degree, with *d*s in the range of [−0.27, −0.22]. Sexual minority participants presented higher scores in all three dimensions of OSA, with effects ranging from *d* = 0.48 for the Isolated to *d* = 0.94 for the Social. All *p*s < 0.001.

#### Association Between OSA and Off-Line Sexual Behavior

In general, the correlations between the different types of OSA and the variables related to vaginal and anal sex were small, *M*_|r|_ (mean unsigned correlation) = 0.11. Those relations were larger for anal behavior, *M*_|r|_ = 0.15 than for vaginal behavior, *M*_|r|_ = 0.07. Social activity was more closely related to off-line behavior, *M*_|r|_ = 0.15 than were Compulsive or Isolated behaviors, *M*_|r|_ = 0.11 and *M*_|r|_ = 0.09, respectively. For the seven considered variables of vaginal and anal behavior, the range of mean unsigned correlations with OSA ranged from *M*_|r|_ = 0.23 for number of partners to *M*_|r|_ = 0.04 for proportion of sexual intercourses in the last 3 months with alcohol consumption. The associations with masturbation were much larger, with a positive correlation between online sex behavior and masturbatory frequency, ranging from *r* = 0.41 for Social to *r* = 0.52 for Isolated activity.

#### Association Between OSA and Psychosexual Variables

Overall, the correlations between the different types of OSA and the psychosexual variables were small, although larger than the correlations with off-line behavior, ranging from *M*_|r|_ = 0.21 for Isolated activity to *M*_|r|_ = 0.25 for Social activity. The largest correlations for the three factors of OSA were with SOI-R Desire, *M*_|r|_ = 0.41, followed by SSS Preoccupation, *M*_|r|_ = 0.31. The smallest correlations were with SSS Self-Esteem as Sexual Partner, *M*_|r|_ = 0.04, followed by SSS Dissatisfaction with Sexual Life, *M*_|r|_ = 0.15.

### Hierarchical Regression Models

#### Models Relating OSA and Off-Line Sexual Behavior

These results can be seen in Table 3. Controlling for sex, relational status, sexual orientation, and the other two types of online sexual behavior, the only factor that presented statistically significant regression coefficients was Social OSA. To interpret the results, we must consider the interpretation of the intercept in the different models: The expected value in the criteria for a heterosexual woman not involved in a relationship with a score equal to the sample mean in Compulsive, Isolated, and Social OSA. An increment of one standard deviation in Social OSA, controlling for the other variables, was related to: (a) a reduction of 0.36 years in the age of vaginal sex debut, *p* <0.001, and 0.39 years in the age of anal sex debut, *p* = 0.017, while the intercepts were 16.93 and 19.00 years, respectively; (b) an increment of 1.83 sexual partners in the lifetime for vaginal behavior, *p* < 0.001, and 0.46 partners for anal behavior, *p* < 0.001, while the intercepts were 4.43 and 0.30 partners; (c) an increment of 0.22 sexual partners in the last 3 months for vaginal behavior, *p* < 0.001, and of 0.08 partners for anal behavior, *p* < 0.001, while the intercepts were 0.84 and 0.06 partners; and (d) an increment of 2.06 vaginal relationships in the last 3 months, *p* = 0.005, and of 0.30 anal relationships, *p* = 0.011, while the intercepts were 6.18 and 0.08 relationships. Masturbation frequency was an exception, where all three OSA presented statistically significant regression coefficients, *b* = 0.21 (*p* = 0.010) for Compulsive use, *b* = 0.45 (*p* < 0.001) for Isolated activity, and 0.23 (*p* = 0.001) for Social use, while the intercept was 1.66 times/week. OSA scores was did not present statistically significant coefficients with risky behaviors, with the exception of Social activity with respect to vaginal sex under the effects of alcohol consumption, *b* = 0.02, *p* = 0.013, with an intercept equal to 0.08.

**Table 3:** Hierarchical regressions of vaginal and anal sex behavior variables predicted from the variables evaluated.

	**Age of onset**			**Partners**			**Partners (3 months)**			**Relations (3 months)**			**Prop. condom use**			**Prop. sex with alcohol**			**Prop. sex with drugs**					
***n***	**979**			**1,100**			**1,095**			**1,089**			**788**			**790**			**799**					
	**Δ*****R***^**2**^	**Δ*****F***	***p***	**Δ*****R***^**2**^	**Δ*****F***	***p***	**Δ*****R***^**2**^	**Δ*****F***	***p***	**Δ*****R***^**2**^	**Δ*****F***	***p***	**Δ*****R***^**2**^	**Δ*****F***	***p***	**Δ*****R***^**2**^	**Δ*****F***	***p***	**Δ*****R***^**2**^	**Δ*****F***	***p***			
**VAGINAL SEXUAL BEHAVIOR**																								
Block 1	**0.03**	**11.30**	** <0.001**	0.00	0.25	0.863	**0.02**	**7.90**	** <0.001**	**0.18**	**79.03**	** <0.001**	**0.02**	**4.66**	**0.003**	**0.17**	**52.35**	** <0.001**	**0.01**	**3.76**	**0.011**			
Block 2	**0.02**	**8.61**	** <0.001**	**0.09**	**34.26**	** <0.001**	**0.06**	**21.63**	** <0.001**	**0.01**	**5.17**	**0.002**	0.00	1.04	0.373	0.00	0.23	0.877	**0.02**	**6.87**	** <0.001**			
	***b***	**SE**	***p***	***b***	**SE**	***p***	***b***	**SE**	***p***	***b***	**SE**	***p***	***b***	**SE**	***p***	***b***	**SE**	***p***	***b***	**SE**	***p***			
Intercept	**16.93**	**0.10**	** <0.001**	**4.43**	**0.28**	** <0.001**	**0.84**	**0.04**	** <0.001**	**6.18**	**0.98**	** <0.001**	**0.69**	**0.03**	** <0.001**	**0.30**	**0.02**	** <0.001**	**0.08**	**0.01**	** <0.001**			
Men	**0.50**	**0.14**	** <0.001**	**−1.09**	**0.39**	**0.005**	**−0.22**	**0.06**	** <0.001**	**−4.56**	**1.36**	**0.001**	0.02	0.04	0.631	−0.03	0.02	0.155	**−0.05**	**0.02**	**0.001**			
Relation	**−0.30**	**0.11**	**0.005**	0.11	0.31	0.731	**0.22**	**0.05**	** <0.001**	**16.31**	**1.08**	** <0.001**	**−0.10**	**0.03**	**0.002**	**−0.21**	**0.02**	** <0.001**	−0.03	0.01	0.051			
Minority	**−0.35**	**0.17**	**0.042**	**−1.10**	**0.49**	**0.024**	**−0.28**	**0.08**	** <0.001**	**−3.66**	**1.69**	**0.031**	−0.09	0.05	0.070	0.02	0.03	0.500	0.01	0.02	0.698			
COM	0.12	0.09	0.181	−0.33	0.26	0.199	−0.04	0.04	0.266	0.36	0.89	0.686	−0.04	0.03	0.104	0.00	0.01	0.879	0.01	0.01	0.466			
ISO	0.07	0.07	0.353	0.09	0.21	0.685	0.03	0.03	0.334	0.00	0.73	0.998	0.03	0.02	0.247	0.00	0.01	0.967	0.01	0.01	0.330			
SOC	**−0.36**	**0.07**	** <0.001**	**1.83**	**0.21**	** <0.001**	**0.22**	**0.03**	** <0.001**	**2.06**	**0.74**	**0.005**	0.01	0.02	0.727	0.01	0.01	0.465	**0.02**	**0.01**	**0.013**			
**ANAL SEXUAL BEHAVIOR**																						**MASTURBATION**		
	**Age of onset**			**Partners**			**Partners (3 months)**			**Relations (3 months)**			**Prop. condom use**			**Prop. sex with alcohol**			**Prop. sex with drugs**					
***n***	**266**			**1,094**			**1,092**			**1,092**			**116**			**117**			**117**			**1077**		
	**Δ**R**^2^**	**Δ*****F***	***p***	**Δ**R**^2^**	**Δ*****F***	***p***	**Δ*****R***^**2**^***R***	**Δ*****F***	***p***	**Δ*****R***^**2**^	**Δ*****F***	***p***	**Δ*****R***^**2**^***R***	**Δ*****F***	***p***	**Δ*****R***^**2**^	**Δ*****F***	***p***	**Δ*****R***^**2**^	**Δ*****F***	***p***	**Δ**R**^2^**	**Δ*****F***	***p***
Block 1	**0.09**	**9.02**	** <0.001**	**0.09**	**36.41**	** <0.001**	**0.05**	**19.97**	** <0.001**	**0.05**	**19.59**	** <0.001**	0.06	2.57	0.058	0.03	1.09	0.358	0.01	0.41	0.745	**0.33**	**174.04**	** <0.001**
Block 2	0.03	2.49	0.061	**0.04**	**16.49**	** <0.001**	**0.05**	**18.53**	** <0.001**	**0.02**	**8.25**	** <0.001**	0.04	1.71	0.169	0.00	0.17	0.915	**0.07**	**2.76**	**0.046**	**0.10**	**59.58**	** <0.001**
	***b***	**SE**	***p***	***b***	**SE**	***p***	***b***	**SE**	***p***	***b***	**SE**	***p***	***b***	**SE**	***p***	***b***	**SE**	***p***	***b***	**SE**	***p***	***b***	**SE**	***p***
Intercept	**19.00**	**0.22**	** <0.001**	**0.30**	**0.11**	**0.008**	**0.06**	**0.02**	**0.004**	0.08	0.15	0.601	**0.31**	**0.07**	** <0.001**	**0.24**	**0.05**	** <0.001**	**0.15**	**0.05**	**0.003**	1.66	0.09	** <0.001**
Men	−0.25	0.30	0.414	**0.32**	**0.15**	**0.037**	0.00	0.03	0.888	0.00	0.21	0.984	0.18	0.10	0.073	−0.07	0.07	0.366	**−0.14**	**0.07**	**0.040**	1.50	0.12	** <0.001**
Relation	0.31	0.24	0.201	−0.06	0.12	0.608	**0.07**	**0.02**	**0.002**	**0.43**	**0.17**	**0.012**	−0.14	0.08	0.088	−0.04	0.06	0.481	0.00	0.05	0.998	−0.45	0.10	** <0.001**
Minority	**−1.39**	**0.38**	** <0.001**	**1.30**	**0.19**	** <0.001**	**0.15**	**0.03**	** <0.001**	**1.40**	**0.27**	** <0.001**	**0.30**	**0.13**	**0.018**	−0.14	0.09	0.149	0.00	0.08	0.992	0.25	0.15	0.102
COM	0.22	0.20	0.275	0.00	0.10	0.997	−0.01	0.02	0.570	0.14	0.14	0.336	−0.05	0.07	0.438	−0.01	0.05	0.887	0.03	0.04	0.458	**0.21**	**0.08**	**0.010**
ISO	−0.14	0.16	0.373	−0.01	0.08	0.934	0.03	0.01	0.077	0.13	0.12	0.272	−0.07	0.05	0.196	0.03	0.04	0.492	0.05	0.04	0.207	**0.45**	**0.07**	** <0.001**
SOC	**−0.39**	**0.16**	**0.017**	**0.46**	**0.08**	** <0.001**	**0.08**	**0.01**	** <0.001**	**0.30**	**0.12**	**0.011**	0.01	0.06	0.869	0.00	0.04	0.931	0.03	0.04	0.470	**0.23**	**0.07**	** <0.001**

*Men is a dummy variable where 0 = woman and 1 = man. Relation is a dummy variable where 0 = not in a romantic relationship and 1 = in a romantic relationship. Minority is a dummy variable where 0 = heterosexual and 1 = sexual minority. COM, Compulsive use; ISO, Isolated use; SOC, Social participation; Prop., Proportion of sexual relationships. Masturbation in times per week. Bold values correspond to statistically significant results*.

#### Models Relating OSA and Psychosexual Variables

These results can be seen in Table 4. In these models, the interpretation of the regression coefficients is different from the previous models, as now the criteria variables were standardized (mean and standard deviation from the total sample). So, now, the coefficients indicate the expected change in the standardized criteria. Social activity scores had statistically significant coefficients with all the considered variables: It was related to higher self-esteem as sexual partner, *b* = 0.19, *p* < 0.001; lower sexual dissatisfaction, *b* = −0.10, *p* = 0.014; higher sexual preoccupation, *b* = 0.12, *p* = 0.003; higher sociosexual behavior, *b* = 0.45, *p* < 0.001; more positive attitudes about casual sex, *b* = 0.30, *p* < 0.001; higher sociosexual desire, *b* = 0.20, *p* < 0.001; and fewer sexual problems, *b* = −0.09, *p* = 0.025. Compulsive use scores presented a different pattern of statistically significant coefficients. Increments in Compulsive behavior were related to lower self-esteem as sexual partner, *b* = −0.14, *p* = 0.005; higher dissatisfaction with sexual life, *b* = 0.16, *p* = 0.001; higher sexual preoccupation, *b* = 0.18, *p* < 0.001; worse attitudes toward casual sex, *b* = −0.12, *p* = 0.014; and higher desire for casual sex, *b* = 0.09, *p* = 0.032. Isolated activity scores presented less statistically significant coefficients. Increments of Isolated use were associated with higher preoccupation, *b* = 0.08, *p* = 0.045; better attitudes toward casual sex, *b* = 0.12, *p* = 0.002; higher desire for casual sex, *b* = 0.11, *p* = 0.001; and better sexual functioning, *b* = −0.12, *p* = 0.003. As can be inferred from this description, the mean unsigned *b* coefficient was greater for Social participation (*M*_|b|_ = 0.21) than for Compulsive (*M*_|b|_ = 0.11) or Isolated (*M*_|b|_ = 0.08) activity.

**Table 4:** Hierarchical regressions of predicted psychosexual variables based on sociodemographic variables and OSA scores.

	**SSS Self–esteem**			**SSS Dissatisfaction**			**SSS Preoccupation**			**SOI–R Behavior**			**SOI–R Attitudes**			**SOI–R Desire**			**ASEX Sexual function**^****a****^		
***n***	**1,044**			**1,055**			**1,092**			**1,069**			**1,094**			**1,090**			**1,034**		
	**Δ*****R***^**2**^	**Δ*****F***	***p***	**Δ*****R***^**2**^	**Δ*****F***	***p***	**Δ*****R***^**2**^	**Δ*****F***	***p***	**Δ*****R***^**2**^	**Δ*****F***	***p***	**Δ*****R***^**2**^	**Δ*****F***	***p***	**Δ*****R***^**2**^	**Δ*****F***	***p***	**Δ*****R***^**2**^	**Δ*****F***	***p***
**PSYCHOSEXUALITY**																					
Block 1	**0.06**	**2.75**	** <0.001**	**0.18**	**76.02**	** <0.001**	**0.06**	**24.96**	** <0.001**	**0.02**	**8.62**	** <0.001**	**0.04**	**16.27**	** <0.001**	**0.27**	**132.54**	** <0.001**	**0.10**	**37.21**	** <0.001**
Block 2	**0.02**	**8.12**	** <0.001**	**0.01**	**5.85**	**0.001**	**0.08**	**31.72**	** <0.001**	**0.14**	**59.30**	** <0.001**	**0.07**	**27.59**	** <0.001**	**0.09**	**48.02**	** <0.001**	**0.02**	**7.57**	** <0.001**
	***b***	**SE**	***p***	***b***	**SE**	***p***	***b***	**SE**	***p***	***b***	**SE**	***p***	***b***	**SE**	***p***	***b***	**SE**	***p***	***b***	**SE**	***p***
Intercept	**−0.26**	**0.06**	** <0.001**	**0.41**	**0.05**	** <0.001**	−0.04	0.05	0.476	**0.11**	**0.05**	**0.028**	0.03	0.05	0.594	**0.28**	**0.05**	** <0.001**	**0.38**	**0.05**	** <0.001**
Men	−0.06	0.08	0.410	**0.16**	**0.07**	**0.027**	**0.17**	**0.07**	**0.019**	−0.11	0.07	0.129	0.11	0.07	0.125	**0.37**	**0.06**	** <0.001**	**−0.41**	**0.08**	** <0.001**
Relation	**0.49**	**0.06**	** <0.001**	**−0.77**	**0.06**	** <0.001**	−0.02	0.06	0.725	**−0.14**	**0.06**	**0.019**	**−0.13**	**0.06**	**0.027**	**−0.67**	**0.05**	** <0.001**	**−0.40**	**0.06**	** <0.001**
Minority	−0.10	0.10	0.307	0.02	0.09	0.859	−0.01	0.09	0.879	−0.01	0.09	0.949	0.13	0.09	0.174	0.04	0.08	0.619	−0.14	0.09	0.133
COM	**−0.14**	**0.05**	**0.005**	**0.16**	**0.05**	**0.001**	**0.18**	**0.05**	** <0.001**	−0.07	0.05	0.135	**−0.12**	**0.05**	**0.014**	**0.09**	**0.04**	**0.032**	0.03	0.05	0.567
ISO	0.07	0.04	0.102	0.03	0.04	0.471	**0.08**	**0.04**	**0.045**	0.00	0.04	0.911	**0.12**	**0.04**	**0.002**	**0.11**	**0.03**	**0.001**	**−0.12**	**0.04**	**0.003**
SOC	**0.19**	**0.04**	** <0.001**	**−0.10**	**0.04**	**0.014**	**0.12**	**0.04**	**0.003**	**0.45**	**0.04**	** <0.001**	**0.30**	**0.04**	** <0.001**	**0.20**	**0.03**	** <0.001**	**−0.09**	**0.04**	**0.025**

*Men is a dummy variable where 0 = woman and 1 = man. Relation is a dummy variable where 0 = not in a romantic relationship and 1 = in a romantic relationship. Minority is a dummy variable where 0 = heterosexual and 1 = sexual minority. COM, Compulsive use; ISO, Isolated use; SOC, Social participation; SSS, Short version of the Sexuality Scale; SOI-R, Sociosexual Orientation Inventory-Revised; ASEX, Arizona Sexual Experience Scale. Bold values correspond to statistically significant results*.

a *In the ASEX, higher scores indicate lower sexual functioning*.

## Discussion

Comprehension of OSA is important for researchers of sexuality, as it is a phenomenon that, in just two decades, has acquired great relevance worldwide. Due to its recency, the existing literature in this field has some limitations, as we noted in the Introduction. In order to provide useful information, the main objective of this study was to analyze the relationships between the various types of OSA (compulsive, isolated, and social), offline sexual behavior, and a series of psychosexual variables that are relevant to the well-being of the individual. In addition, we intended to improve our knowledge of the psychometric properties of the ISST (Delmonico and Miller, 2003; Ballester-Arnal et al., 2010).

The first contribution of the study, therefore, involves the measuring instrument. The ISST was chosen (Delmonico and Miller, 2003) because some reviews highlighted it as one of the main questionnaires to assess the use of OSA. However, both the original version and the Spanish adaptation (Ballester-Arnal et al., 2010) had some limitations. Through this study, we have tried to overcome them. We have performed a new validation, working with factor scores, proposing a new composition of the scale and contributing to its psychometric properties. Moreover, we have found the existence of three dimensions of the questionnaire (Compulsive, Isolated, and Social), and two items that enrich and update the instrument have been included. However, it is still necessary to create and validate new tools, more up-to-date and with better psychometric properties, to assess OSA. We recommend that the ISST based on observed scores should cease to be used. Future uses of the ISST should be based on structural equation modeling or factor scores, although low conditional reliabilities were found for low factor scores in all dimensions.

As in previous studies with university students, we found that men and sexual minority participants reported greater use of online sex (Shaughnessy et al., 2011b; Ballester-Arnal et al., 2017; Giordano and Cashwell, 2017), although the difference between men and women was lower for social participation. Therefore, it can be concluded that men use online sex as a complement and a substitute for offline sex, regardless of the type and whether or not they have a partner (Ballester-Arnal et al., 2017). The same applies to sexual minority participants [see (Döring, 2009)]. For them, it seems that Internet provides a reliable and anonymous source of contacts and relationships, especially in contexts that are restrictive toward certain sexual orientations (Daneback et al., 2005; Chaney and Blalock, 2006).

Greater use of online sex was also found in participants who had no partner. The existing literature in this subject is not conclusive. On the one hand, one could think that people without a partner may devote more time to online sex. However, other studies argue that having or not having a partner does not determine this use because many couples use it as a complement of, and even to initiate, offline sex (Daneback et al., 2005; Döring, 2009; Griffiths, 2012; Ballester-Arnal et al., 2017).

This shows the relationship between online and offline sexual behavior. In this section, one of the contributions of the study is having taken into account not only vaginal behavior, but also anal behavior, due to the high usage by sexual minority people (Ross and Kauth, 2002; Chaney and Blalock, 2006), and of masturbation. Although only moderate associations between online and offline sexual behavior were observed, three findings are highlighted. The first is that social online sex had the highest relation with offline sexual behavior, both vaginal and anal, especially with a younger initiation age, a greater number of partners, and a higher proportion of relations under the influence of drugs. This result seems logical because social users have more contacts and more social networks to have offline sex (Ross and Kauth, 2002). If part of social OSA consists of finding sexual partners, it is reasonable that more use implies more partners. Compulsive and solitary uses are only related to a younger age of initiation in anal relationships, a greater number of partners, and a higher proportion of vaginal relations under the influence of drugs.

The second finding is that no relationship was found between OSA, regardless of the modality, and the use of condoms, either in anal or vaginal relations. This is positive because it does not support the idea that online sex increases vulnerability to sexually transmitted infections (Liau et al., 2006). Finally, a relationship was found between the three types of OSA and frequency of masturbation, which underscores the preference of many participants, especially men, for solitary consumption of pornography (Wéry and Billieux, 2017).

Another important contribution of the study is the analysis of the associations between different types of OSA and the psychosexual variables evaluated. Three main conclusions can be drawn. The first is that some associations could change their sign when considered in bivariate models or in multiple regressions. For instance, Compulsive behavior had positive and statistically significant correlations with number of sexual partners, but statistically non-significant negative or zero coefficients when controlling for the other variables. The second conclusion is that the three types of OSA relate differentially to psychosexual well-being. This second points stresses that not all OSA are the same. The third conclusion is that most young people use healthy online sex, both solitary and social, as they are related –especially the latter– to psychosexual well-being (Ballester-Arnal et al., 2017). Social participation was associated with higher self-esteem as a sexual partner, more satisfaction with sex life, more thoughts about sex, more socio-sexuality—in its three components—and better sexual performance. The correlates of solitary use were similar. Therefore, OSA can have positive consequences and affect the individual's well-being.

As for compulsive use, it was related to lower satisfaction with sex life and more preoccupation with sex, in addition to higher sociosexuality and lower self-esteem as a sexual partner, although with very low and non-significant coefficients. These associations are not too different from those of the other types of uses and, above all, not as adverse as those of other studies that have linked the problematic use of online sex to general measures, such as anxiety, depression, low self-esteem, or relational difficulties (Kor et al., 2014; Harper and Hodgins, 2016). However, there is still much progress to be made in the study of the relationship of the three types of uses and psychosexual well-being, first clarifying the diagnostic criteria and guidelines, determining their correlates, and designing effective intervention programs.

The study has a series of limitations, primarily related to the composition and representativeness of the sample, which hinders the generalizability of the results. First, the different inclusion criteria led to a large reduction of participants. As explained, this can be explained by the fact that the ISST was the last questionnaire in a larger battery and many participants did not reach the final items. Among those who provided responses to the ISST, the sample was mostly female and heterosexual. In addition, as the sample came from a single university, and we restricted age range, it cannot be said that the results are generalizable to all university students and still less to non-university youth between ages 18 and 26 years. Thirdly, our study shares with other studies based on self-selected samples and self-reported measures the fact that the results may be limited by response and recall bias. Finally, it has some limitations that are typical of the literature on OSA, such as the difficulty to define the construct and encompass all the activities it involves and the speed of changes in activities and behaviors that easily outdate the instruments and the dimensions evaluated.

Despite these limitations, we consider that the results of the study provide relevant information for research of OSA. It offers a new validation and improvement of the ISST, the first to employ up-to-date psychometric techniques. It has differentiated between three dimensions of OSA. We have made clear the appropriateness of controlling for the other types of uses when evaluating each dimension. We have developed a socio-demographic and psychosexual profile of OSA users and have analyzed it with offline sexual behavior, not just vaginal, but also anal and masturbatory. In addition, we have highlighted the healthy use of online sex and its relationship with psychosexual well-being. Thus, it is important to continue investigating in this field and to implement programs of psychosexual health promotion and prevention of problems, targeting young people in order to promote the healthy use and develop specific skills to combat risky sexual behavior and problematic use of online sex.

## Data Availability

The raw data supporting the conclusions of this manuscript will be made available by the authors, without undue reservations, to any qualified researchers.

## Ethics Statement

This study was carried out in accordance with the recommendations of the ethics research committee of the Spanis region of Aragón (Comité de Ética para la Investigación Clínica de Aragón-Ethics Committee for Clinical Research of Aragon), with written informed consent from all subjects. All subjects gave written informed consent in accordance with the Declaration of Helsinki. The protocol was approved by the Ethics Committee for Clinical Research of Aragon (Spain).

## Author Contributions

ÁC and JB contributed conception and design of the study and wrote the final version of the manuscript. PR-G did bibliographic search and wrote the first draft of the manuscript. JB organized the database and performed the statistical analysis. ÁC reviewed the first draft of the manuscript. PR-G and ABC wrote the second draft of the manuscript. All authors contributed to manuscript revision, read and approved the submitted version.

### Conflict of Interest Statement

The authors declare that the research was conducted in the absence of any commercial or financial relationships that could be construed as a potential conflict of interest.
